# SOX7 promotes the maintenance and proliferation of B cell precursor acute lymphoblastic cells

**DOI:** 10.18632/oncotarget.10472

**Published:** 2016-07-07

**Authors:** Sara Cuvertino, Genny Filiciotto, Ashish Masurekar, Vaskar Saha, Georges Lacaud, Valerie Kouskoff

**Affiliations:** ^1^ Cancer Research UK Manchester Institute, The University of Manchester, Manchester M20 4BX, UK; ^2^ Children’s Cancer Group, Institute of Cancer Sciences, The University of Manchester, Manchester M20 4BX, UK; ^3^ TCS Translational Cancer Research Centre, Tata Medical Centre, Kolkata 700156, India; ^4^ Present address: Genetic Medicine, Institute of Human Development, University of Manchester, St Mary’s Hospital, Manchester M13 9WL, UK

**Keywords:** SOX7, leukemia, B-ALL

## Abstract

B cell precursor acute lymphoblastic leukemia (BCP-ALL) is the most frequent type of cancer in children. Despite progresses in curative treatment, intensive chemotherapy regimens still cause life threatening complications. A better understanding of the molecular mechanisms underlying the emergence and maintenance of BCP-ALL is fundamental for the development of novel therapies. Here, we establish that *SOX7* is frequently and specifically expressed in BCP-ALL and that the expression of this transcription factor does not correlate with any specific cytogenetic abnormalities. Using human leukemia model systems, we establish that the down-regulation of *SOX7* in BCP-ALL causes a significant decrease in proliferation and clonogenicity *in vitro* that correlates with a delay in leukemia initiation and burden *in vivo*. Overall, these results identify a novel and important functional role for the transcription factor SOX7 in promoting the maintenance of BCP-ALL.

## INTRODUCTION

Acute lymphoblastic leukemia (ALL) is the most frequent type of cancer in children and comprises neoplastic precursor cells committed to the B cell (BCP-ALL) or the T cell (T-ALL) lineages. BCP-ALL represents the majority of ALL, accounting for up to 85% of childhood ALL and 75% of adult ALL [1]. Genes involved in B cell development such as *EBF1*, *IKZF1*, *PAX5* or *PBX1* are frequently found mutated in BCP-ALL [2, 3]. Additionally, cytogenetic abnormalities are frequently detected and often define specific subtypes of the disease with unique prognostic features. While childhood BCP-ALL is curable in most cases with a survival rate approaching 80%, the rate of cure for adult patients presenting with BCP-ALL is only 40% [4, 5]. Despite progresses in the treatment of BCP-ALL, intensive chemotherapy regimens cause life threatening complication and around 20% of the treated patients relapse presenting then a very poor outcome [6]. There is a great need for the development of less toxic compounds and novel therapies for the treatment of relapsed and specific subgroups of BCP-ALL patients with poor prognosis.

The SOX family of transcription factor comprises 20 members divided into subgroups based on sequence similarities [7]. SOX factors are essential for the maintenance of various stem cell compartments during embryogenesis and adult life, such as for example SOX2 in neuronal stem progenitor cells [8] or SOX17 in fetal hematopoietic stem cells [9]. In addition, evidences for the involvement of SOX factors in cancers, either as tumour suppressors or as proto-oncogenes, have emerged recently. Deregulation of *SOX* gene expressions have been widely documented in cancer [10], and a few studies have identified a direct involvement of SOX factors in tumorigenesis [11–14]. SOX7 and its close homologues, SOX17 and SOX18, belong to the SOX F subgroup and play important roles in cardiovascular development [15–17]. The SOX F factors are also involved in the development of the hematopoietic system with SOX17 driving the expansion of fetal hematopoietic stem cells [9] while SOX7 and SOX18 are expressed at the earliest stages of blood development [18, 19]. The expression of *SOX7* is frequently down-regulated in solid tumours such as prostate, colon and endometrial cancers, but the functional relevance of these findings remain unknown [20, 21]. A recent study has also revealed a tumor suppressor role for SOX7 in acute myeloid leukemia (AML) [22]. In the present study, we establish that SOX7 is frequently expressed in BCP-ALL and that the expression of this transcription factor is critical for BCP-ALL proliferation and maintenance.

## RESULTS

### *SOX7* is frequently over-expressed in human BCP-ALL

**Figure 1:**
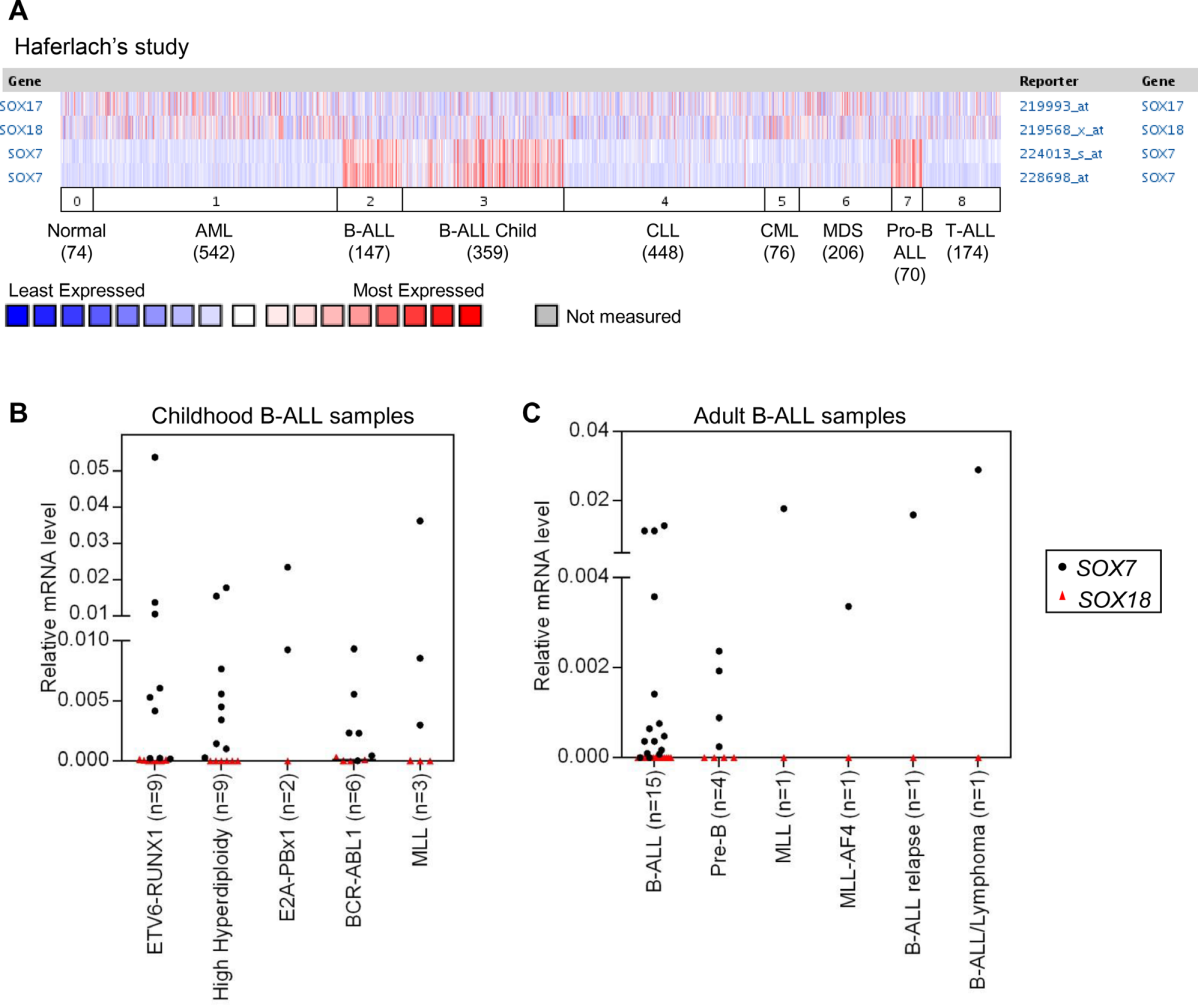
*SOX7* is specifically expressed in human BCP-ALL samples **A.** *SOX7* expression dataset of human leukemia samples obtained from the Oncomine database. 0. Normal samples (74), 1. Acute Myeloid Leukemia (AML; 542), 2. B-Cell Acute Lymphoblastic Leukemia (BCP-ALL; 147), 3. B-Cell Childhood Acute Lymphoblastic Leukemia (BCP-ALL child; 359), 4. Chronic Lymphocytic Leukemia (CLL; 448), 5. Chronic Myelogenous Leukemia (CML; 76), 6. Myelodysplastic Syndrome (MDS; 206), 7. Pro-B Acute Lymphoblastic Leukemia (Pro BCP-ALL; 70), 8. T-Cell Acute Lymphoblastic Leukemia (T-ALL; 174). Two Affymetrix probes are shown for *SOX7*. **B.** Quantitative RT-PCR analysis of *SOX7* and *SOX18* transcript levels relative to β2-microglobulin in childhood human BCP-ALL samples (bone marrow). **C.** Quantitative RT-PCR analysis of *SOX7* and *SOX18* transcript levels relative to β2microglobulin in adult human BCP-ALL samples (blood).

To determine whether *SOX7* expression was associated with hematological malignancies, we interrogated the Oncomine database for differential *SOX7* expression in leukemias. This analysis revealed that *SOX7* expression levels were frequently and significantly higher in childhood and adult BCP-ALL when compared to normal bone marrow and other types of leukemia, including T-ALL, acute myeloid leukemia (AML), chronic leukemias and myelodysplastic syndrome (MDS) (Figure 1A and S1A–S1B). In contrast, the expression of *SOX17* and *SOX18* did not show any specific or restricted pattern of expression in any hematological malignancies (Figure 1A). To validate this observation, the expression of *SOX7* and *SOX18* was evaluated in a small cohort of human ALL samples (Figure 1B–1C). These analyses confirmed that whilst *SOX7* expression was high in multiple cases of BCP-ALL, *SOX18* expression remained undetectable. Overall, *SOX7* expression did not correlate with any specific type of chromosomal translocation or mutation known to be involved in BCP-ALL as confirmed by further analysis of the Oncomine datasets (Supplementary Figure S1C-S1D). Altogether, these results establish that the transcription factor *SOX7* is specifically and frequently expressed in human BCP-ALL, without an obvious association with specific chromosomal abnormalities or cytogenetic subtypes.

### *SOX7* knock-down impairs the proliferation and clonogenicity of BCP-ALL cells

**Figure 2:**
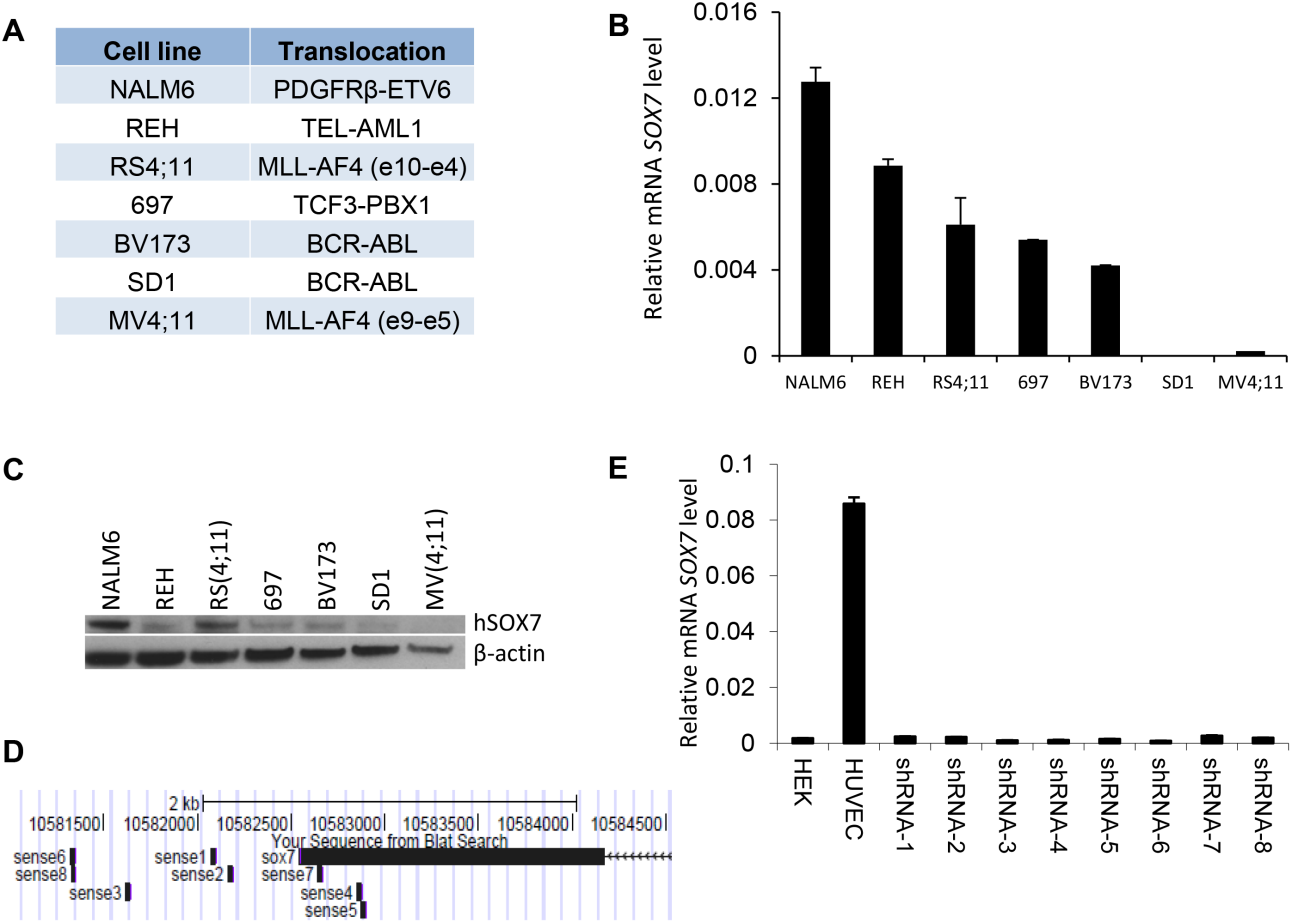
*SOX7* expression in B-ALL cell lines **A.** Human leukemia cell lines and their respective translocations. **B.** Quantitative RT-PCR analysis of *SOX7* transcript level relative to β2microglobulin in leukemia cell lines. Error bars indicate mean ± standard deviation (n=3). **C.** Protein expression level for SOX7 and β-actin for the indicated cell lines measured by Western blot. Data are representative of two independent experiments. **D.** Binding regions of the different shRNAs to the human *SOX7* locus; shRNAs are indicated as sense together with a number. Only shRNA 4, 5 and 7 are binding to *SOX7* coding sequence, all the other shRNAs are binding to the UTR region. The alignment was performed using BLAST browser. **E.** Quantitative RT-PCR analysis of *SOX7* transcript level relative to β2-microglobulin in HUVEC transduced with shRNA against *SOX7* or not. HEK cells were used as negative control. Error bars indicate mean ± standard deviation (n=3).

**Figure 3:**
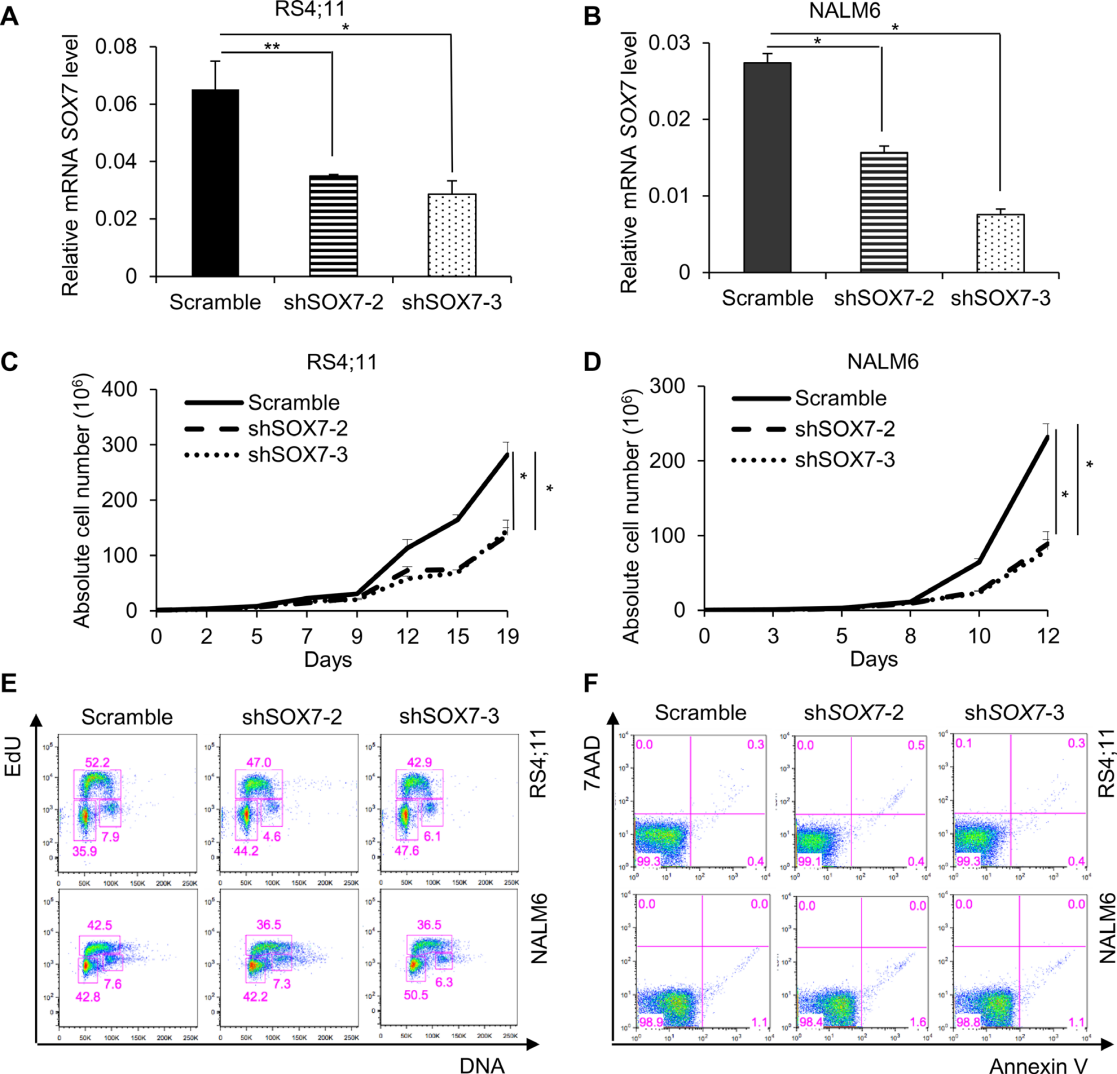
The down-regulation of *SOX7* expression in BCP-ALL cells induces a significant decrease in proliferation **A-B.** Quantitative RT-PCR analysis of *SOX7* transcript levels relative to β2-microglobulin in RS4;11 and NALM6 human leukemia cells transduced with shRNA against *SOX7* or control. Error bars indicate mean ± standard deviation (n=3; * *P*≤ 0.05; ** *P*<0.01). **C-D.** Representative line chart of cell count for RS4;11 and NALM6 cells transduced with shRNA against *SOX7* or control cultured for 19 or 12 days respectively (* *P*<0.05). **E.** Cell cycle status of RS4;11 and NALM6 cells transduced with two different shRNAs against *SOX7* (sh*SOX7*-2, sh*SOX7*-3) or control construct (Scramble). Staining was assessed after 2 hours of EdU incubation. Percentages of cells in the G0/G1, S and G2 phases are shown in the indicated gates. FACS plots are representative of two independent experiments. **F.** Representative FACS plots of 7AAD and Annexin-V expression profiles on RS4;11 and NALM6 cells transduced with two different shRNAs against *SOX7* (sh*SOX7*-2, sh*SOX7*-3) or control construct (Scramble). Percentages of cells are shown in the indicated quadrants. FACS plots are representative of two independent experiments.

**Figure 4:**
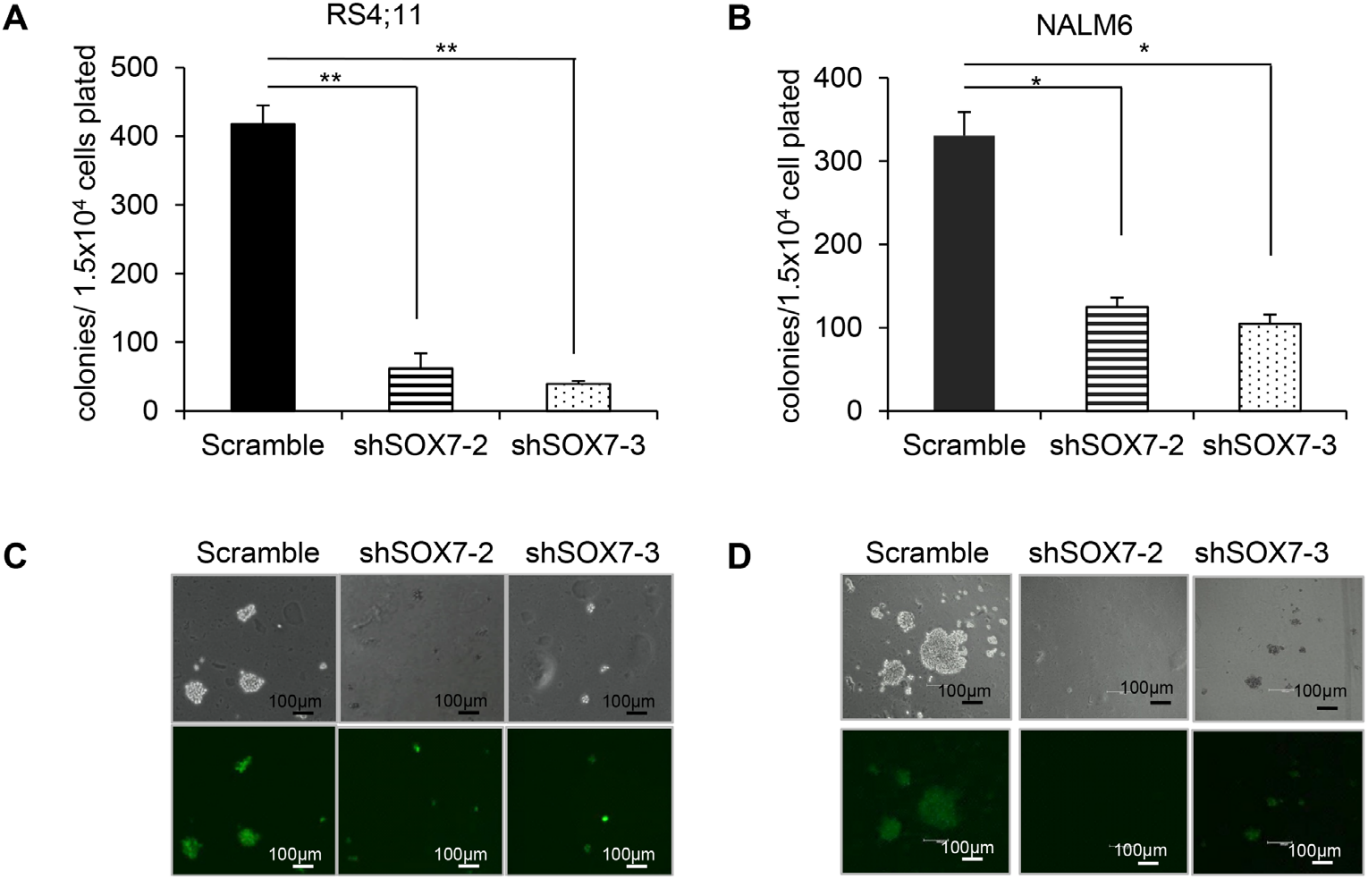
The down-regulation of *SOX7* expression in BCP-ALL cells induces a significant decrease in clonogenicity **A-B.** RS4;11 and NALM6 human leukemia cells transduced with the indicated shRNA were cultured in semi-solid clonogenic assays. Data represent colony number after 10 days in culture (n=3, * *P*<0.05; ** *P*<0.01). **C-D.** Representative pictures of colonies obtained from RS4;11 and NALM6 replating in colony assay at day 10. Scale bars represent 100μm (Leica scan and software). Data are representative of three independent experiments.

To investigate how *SOX7* expression might contribute to the leukemic process, we first analyzed its expression in multiple human BCP-ALL cell lines with different cytogenetic characteristics (Figure 2A). The six lines evaluated expressed varying level of *SOX7* transcript (Figure 2B) and protein (Figure 2C); MV4;11 an AML line used as a negative control did not expressed *SOX7*. To modulate *SOX7* expression, we tested several shRNAs against *SOX7* transcripts (Figure 2D), all of which induced greater than 95% decrease in *SOX7* transcript levels in the endothelial HUVEC cells expressing high level of the *SOX7* transcripts (Figure 2E). However, in BCP-ALL cells, these shRNAs induced much lower levels of knock-down. To evaluate the role of SOX7 in BCP-ALL, we therefore used two shRNAs conferring around 50% decrease in *SOX7* transcript levels in RS4;11 and NALM6 expressing intermediate and high *SOX7* level respectively (Figure 3A–3B). The two *SOX7* shRNAs tested induced a significant decrease in proliferation in both leukemic cell lines when compared to the control shRNA (Figure 3C–3D). The co-transduction of two different SOX7 shRNAs in RS4;11 cells did not further decrease *SOX7* transcript levels or proliferation when compared to cells transduced with a single shRNA (Supplementary Figure S2A-S2B). Cell cycle status via EdU incorporation revealed a reduction in the frequency of cells in the S-phase of the cell cycle for both leukemic cell lines transduced with either shRNAs (Figure 3E). Apoptosis assay showed that BCP-ALL cells were viable without significant increased apoptosis upon *SOX7* knock-down (Figure 3F, positive control of staining Supplementary Figure S3). Next, to test whether *SOX7* down-regulation impacted the clonogenic capacity of the BCP-ALL cells, we cultured the transduced cells in semi-solid colony assay, which represents a more stringent test of clonal proliferative capacity. The down-regulation of *SOX7* by either shRNAs resulted in a significant decrease in colony formation in both leukemia lines (Figure 4A–4B), with much smaller colonies formed upon *SOX7* knock-down (Figure 4C–4D). Altogether, these data demonstrate that the down-regulation of *SOX7* in BCP-ALL human cell lines induced a significant decrease in proliferation, even though *SOX7* transcript levels were only decreased by 50%.

### *SOX7* knock-down delays leukemogenesis *in vivo*

**Figure 5:**
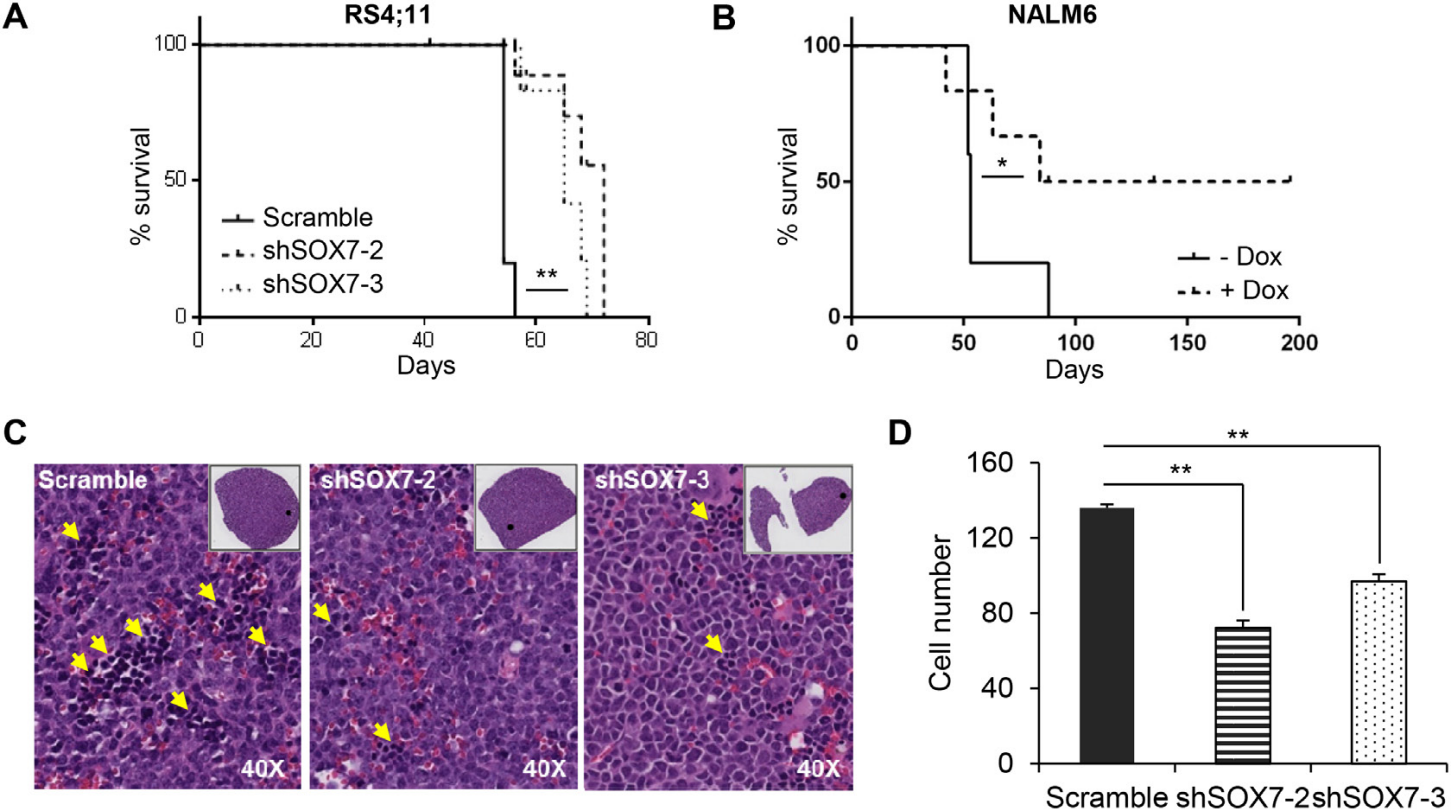
The down-regulation of *SOX7* expression in BCP-ALL cells decreases leukemogenesis burden **A-B.** Kaplan-Meier survival analysis. Mice were injected i.v. with RS4;11 human leukemia cells **A.** transduced with *SOX7* or control shRNA or with NALM6 human leukemia cells **B.** transduced with an inducible *SOX7* shRNA. Statistical differences between groups were calculated using the survival curve statistics Log-rank (Mantel-Cox) test (GraphPad Prism software). RS4;11 data are representative of three independent experiments (** *P*<0.05), NALM6 data represent two experiments (* *P*=0.08). (N=5 mice per group per experiment). **C.** May-Grünwald-Giemsa staining of spleen sections from mice engrafted with RS4;11 human leukemia cells transduced with two different *SOX7* shRNAs (sh*SOX7*-2 and sh*SOX7*-3) or control construct (Scramble). Yellow arrowheads indicate leukemic cells. **D.** Bar chart representing the number of leukemic cells per section. Data are shown as the mean of cell number counted from four different areas in each section. Error bars indicate mean ± standard deviation (n=4, Leica scan and software, magnification: 40X). Data are representative of three independent experiments.

**Figure 6:**
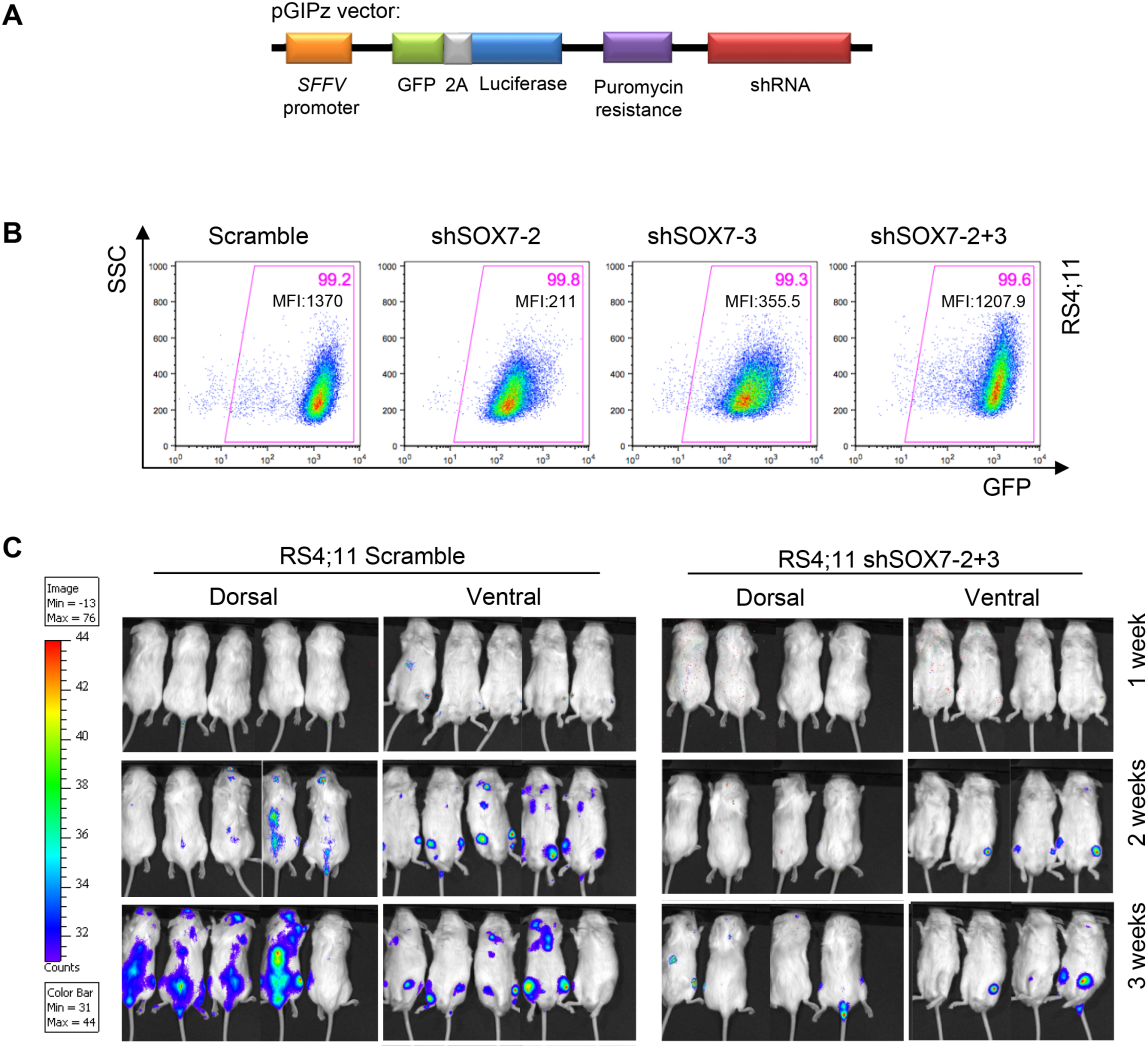
The down-regulation of *SOX7* expression in BCP-ALL cells delays leukemogenesis initiation and spreading **A.** Scheme of the pGIPz lentiviral vector used to transduce the leukemia cell lines. **B.** Representative FACS plots of SSC and GFP expression profiles of RS4;11 human leukemia cells transduced with *SOX7* shRNAs (sh*SOX7*-2, sh*SOX7*-3, sh*SOX7*-2+3) and control construct (Scramble). Percentages of cells and median fluorescent intensities are shown in the indicated quadrants. FACS plots are representative of two independent experiments. **C.** Mice were injected intra-peritoneally with Luciferin and anesthetized before to be imaged (IVIS Lumina, Caliper Life Sciences) for 30 seconds-1 minute per side (dorsal/ventral).

To investigate the role of *SOX7* in the progression of leukemia *in vivo*, recipient mice were engrafted either with RS4;11 leukemia cells transduced with a *SOX7* or control shRNA or with NALM6 leukemia cells transduced with an inducible lentiviral vector encoding a *SOX7* shRNA. An inducible system was tested to determine if acute deletion of *SOX7* transcripts might be more efficient but similar results to the constitutive system were observed (Supplementary Figure S4A-S4B). Upon engraftment of the BCP-ALL control cells, recipient mice progressively developed symptoms indicative of leukemia, such as pale extremities, piloerection, hunched posture and continuous weight loss, ultimately leading to death. The knock-down of *SOX7* expression in both cell lines induced a significant delay in the onset of these symptoms hence resulting in prolonged survival (Figure 5A–5B). A decrease in the leukemic burden was also observed post-mortem in the spleen of recipient mice engrafted with RS4;11 cells in which *SOX7* expression was knocked-down (Figure 5C–5D). To further document leukemia development, *in vivo* imaging was performed for luciferase activity carried along the GFP reporter by the shRNA constructs (Figure 6A). Cells transduced with both sh*SOX7*-2 and sh*SOX7*-3 showed an overall similar level of GFP expression (hence luciferase) than the scramble control cells (Figure 6B), whereas cells transduced with a single *SOX7* shRNA had lower GFP levels (hence luciferase) and therefore were not used for comparative imaging. Non-invasive *in vivo* luciferase imaging at the early stage of the disease clearly revealed a delay in the proliferation of BCP-ALL cells and in the spread of the leukemia through organ infiltration (Figure 6C). Taken together, these results demonstrate that the down-regulation of *SOX7* delays the onset of BCP-ALL and decreases the leukemic burden, establishing the critical contribution of SOX7 expression in maintaining the high proliferative status of the leukemic cells.

## DISCUSSION

While specific recurrent cytogenetic abnormalities and mutations in key transcription factors of B cell lymphopoiesis have been identified in most BCP-ALL cases [1, 2], the molecular mechanisms underlying the emergence and maintenance of this type of leukemia still remain poorly understood. In the present study, we identify SOX7 as a novel and important player in BCP-ALL. We show that *SOX7* is frequently and specifically expressed in BCP-ALL, independently of any specific chromosomal translocation, and that this transcription factor is implicated in the proliferative potential and clonogenicity of leukemic cells. Our study suggests that *SOX7* expression in BCP-ALL is an important factor contributing to leukemia.

The analysis of *SOX7* expression revealed the significant and specific expression of this transcription factor in BCP-ALL but not in other types of blood malignancy or in healthy bone marrow. Expression of *SOX7* in BCP-ALL had no obvious association with specific chromosomal abnormalities or cytogenetic subtypes. Chromosomal translocations frequently found in BCP-ALL such as t(4;11) or t(12;21) producing the MLL-AF4 and ETV6-RUNX1 fusion proteins respectively, are not able on their own to fully recapitulate the human disease, suggesting that other critical events are required to lead to BCP-ALL [23–26], the deregulation of *SOX7* expression could be one of these events. It remains to be determined however how SOX7 expression becomes deregulated, to date no chromosomal translocations or mutations have been identified within the *SOX7* locus. An alternative possibility is that SOX7 is part of the transcriptional machinery regulating B cell development and as a result of mutational events leukemic cells become trapped in a SOX7-expressing stage of B lymphopoiesis in which SOX7 is a critical player of proliferation.

To investigate the role for *SOX7* in leukemogenesis, we knocked-down the expression of *SOX7* in human BCP-ALL cell lines using shRNAs. Eight different shRNA constructs were tested in HUVEC (endothelial cells) and all successfully and efficiently decreased *SOX7* transcripts to less than 5% of the control levels. In contrast, in BCP-ALL cell lines, none of the shRNA constructs was able to decrease *SOX7* transcript levels to less than 50% of the control levels. It is possible that this difference in knockdown efficiency is related to differences in the 3’UTR region of *SOX7* mRNA between HUVEC and BCP-ALL cells as 3’UTR sequences are known to affect mRNA stability and post-transcriptional regulation [27]. Alternatively, it is possible that HUVECs do not rely on SOX7 expression for survival as much as BCP-ALL cell lines as HUVECs also express SOX17 and SOX18 that can control similar gene expression to SOX7 in endothelial cells [16]. Given that BCP-ALL cells do not express the two other SOXF factors, their survival might be compromised by a complete absence of *SOX7* expression. However, even with this partial knock-down of *SOX7*, we observed a marked effect on proliferation and clonogenicity. It is likely that a more complete reduction in *SOX7* level might result in more severe consequences in term of proliferation, survival or differentiation. Attempts to further decrease *SOX7* transcript levels were investigated through transduction with two independent shRNAs or using an inducible shRNA system but none of these attempts was effective at further knocking-down *SOX7* expression. These observations suggest that *SOX7* expression is dispensable for the maintenance of HUVEC cells but that it is most likely critically required for BCP-ALL cell survival and proliferation.

Few SOX7 transcriptional targets have been identified to date and among them, we recently described VE-cadherin as one of SOX7 prime targets during the establishment of the cardiovascular system [28]. Interestingly, the expression of VE-cadherin has been reported in BCP-ALL cell lines and primary samples [29]. Furthermore, down-regulation of VE-Cadherin expression with siRNA or antagonist was shown to induce increase sensitivity to chemotherapy while VE-cadherin over-expression resulted in increased survival upon chemotherapy treatment [30]. Furthermore, the expression of endothelial adhesion molecules such as VE-cadherin has been linked to enhanced migration across the brain-blood endothelial barrier, promoting infiltration of the central nervous system by leukemic cells, an event contributing to relapse and poor outcome [31]. It is possible therefore that part of the leukemogenic potential of SOX7 might be achieved through its direct transcriptional activation of VE-cadherin. It will be important in future work to define the downstream program regulated by SOX7 in BCP-ALL and to identify the key pathways regulating the proliferative potential controlled by SOX7. They might represent potential novel druggable targets for the treatment of poor prognosis and relapsed BCP-ALL. Alternatively, the identification of small molecules that can interfere with SOX7 transcriptional activity either through SOX7-DNA interaction or through protein complex formation will represent interesting avenues of investigation with potential therapeutic benefits.

## MATERIALS AND METHODS

### Cell culture

RS4;11 human leukemia cells were grown in α-MEM (Lonza) supplemented with 10% FCS. NALM6 human leukemia cells were grown in RPMI (Lonza) supplemented with 10% FCS.

### Western blotting

Protein samples were isolated using the RIPA lysis buffer and quantified followed by electrophoresis on 10% NuPAGE Novex Bis-Tris Mini Gels and blotting onto nitrocellulose membranes using Mini iBlot Gel Transfer Stacks Nitrocellulose (Invitrogen) Proteins were detected using human SOX7 antibody (R&D) and β-actin (Sigma).

### Colony forming assay

RS4;11 and NALM6 human leukemia cells were plated in α-MEM or RPMI (both from Lonza) respectively containing 55% methylcellulose (10 g/L), 10% serum (Stem Cell Technology), 10% PFM (Gibco), 2 mM L-Glutamine (Gibco), 180 μg/ml transferrin, 0.5 mM ascorbic acid, 4.5x10^-4^ M MTG, 1% KL, 20 ng/ml IL-3, 10 ng/ml IL-7, 3 ng/ml IL-6 and 10 ng/ml SCF (all from PeproTech). Cultures were maintained at 37°C, 5% CO2. Colonies were counted after 10 days in culture according to their size and morphology.

### Cell cycle analysis

10^6^ cells were incubated with 10 μM EdU for 2 hours, harvested, fixed and permeabilized. EdU detection is based on a reaction between the azide, coupled to Alexa Fluor^®^ 647 dye, and the alkyne in the EdU (Invitrogen). DNA content was measured using Violet406. Acquisition analysis was performed on a LSRII (BD cytometer).

### Transplantation

RS4;11 or NALM6 human leukemia cells were transduced with lentiviruses for 30 minutes at 1200 rpm at MOI10-50. Transduced cells were sorted and transplanted i.v. into sub-lethally irradiated (125cGy) NSG mice. Leukemia growth was assessed by blood analysis, weight and general health monitoring. All animal work was performed under regulations governed by the Home Office Legislation under the Animal Scientific Procedures Act of 1986.

### *In vivo* imaging

Mice were injected intra-peritoneally with Luciferin (75mg/kg; PerkinElmer), anesthetized (3% isofluorane) and imaged (IVIS, Caliper Life Sciences) for 1 minute per side.

### Statistical analyses

Data were analyzed using a Student’s t Test. Significant differences are indicates with * (*P*<0.05) and ** (*P*<0.01). Survival analysis was performed using the Kaplan-Meier method and Log-rank statistical test.

## SUPPLEMENTARY MATERIALS FIGURE


