# Supplementary material for: SOX7 promotes the maintenance and proliferation of B cell precursor acute lymphoblastic cells
Source: Oncotarget. 2016 Jul 7;8(39):64974–83. doi: 10.18632/oncotarget.10472 (PMC5630305; doi:10.18632/oncotarget.10472)
Supplement: Supplementary file 1 [file oncotarget-08-64974-s001.pdf]

SOX7 promotes the maintenance and proliferation of B cell precursor Acute Lymphoblastic cells

SUPPLEMENTARY FIGURES

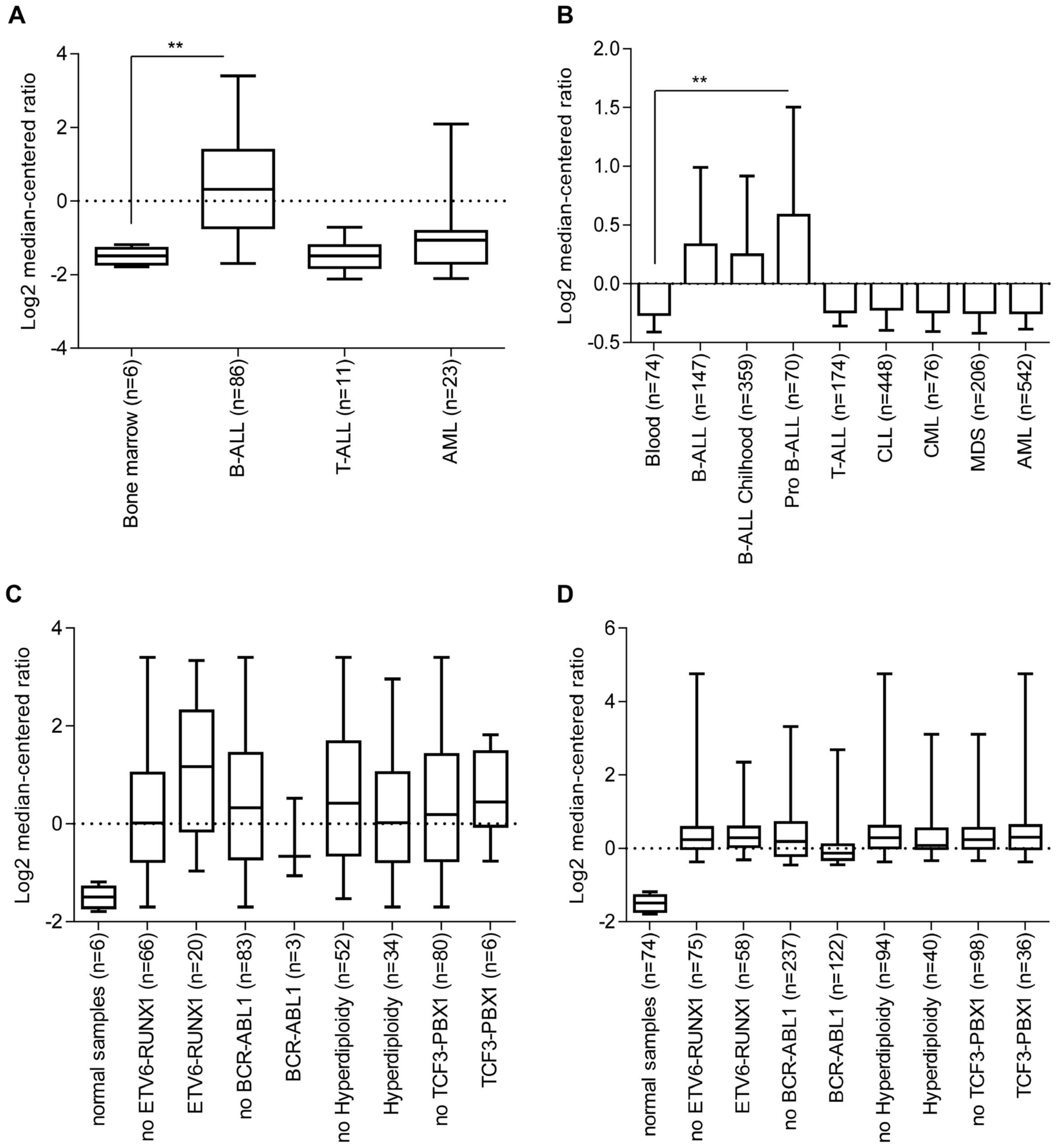

Supplementary Figure S1: *SOX7* is specifically expressed in B-ALL and does not correlate with specific types of mutation involved in B-ALL. A-B. *SOX7* expression in human healthy samples and in different types of leukemias: Anderson study (A) and Haferlach study (B), modified from Oncomine database; ** $P<0.01$. AML: Acute Myeloid Leukemia, B-ALL: B-Cell Acute Lymphoblastic Leukemia, B-ALL Childhood: B-Cell childhood Acute Lymphoblastic Leukemia, CLL: Chronic Lymphocytic Leukemia, CML: Chronic Myeloid Leukemia, MDS: Myelodysplastic Syndrome, Pro B-ALL: Pro-B Acute Lymphoblastic Leukemia, T-ALL: T-Cell Acute Lymphoblastic Leukemia. **C-D.** *SOX7* expression in human B-ALL samples subdivided based on mutations: Anderson study (C) and Haferlach study (D), modified from Oncomine database.

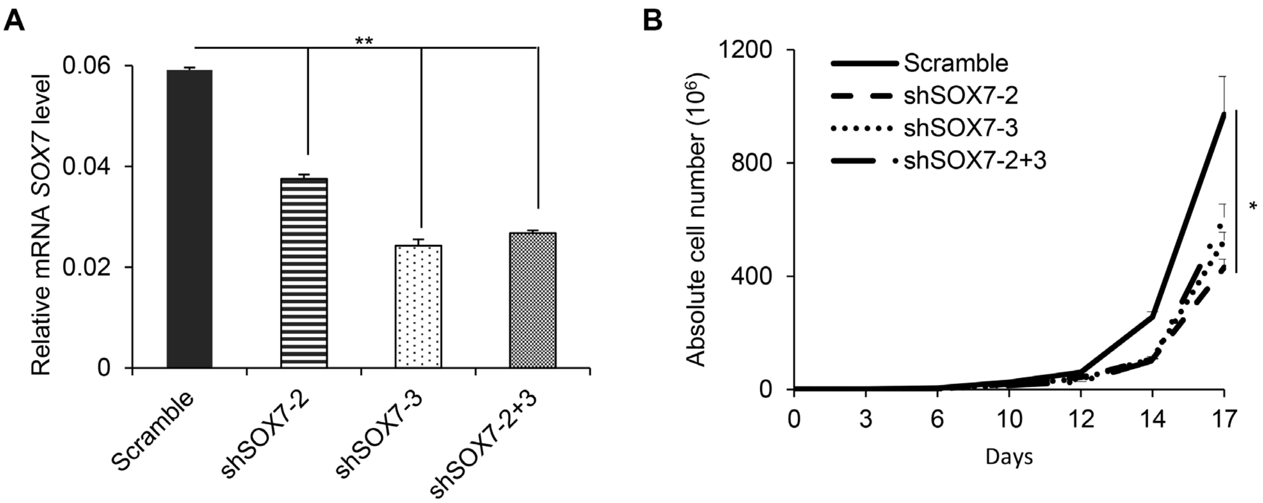

Supplementary Figure S2: Transduction with both shRNAs does not improve the knock-down of *SOX7*. A. Quantitative RT-PCR analysis of *SOX7* transcript level relative to β2microglobulin in RS4;11 human leukemia cells transduced with one or two shRNAs against *SOX7*. Leukemia cells transduced with a control construct were used as a positive control. Error bars indicate mean ± standard deviation (n=3). **B.** Proliferation assay performed with RS4;11 human leukemia cells transduced with one or two shRNAs against *SOX7* (sh*SOX7*-2, sh*SOX7*-3, sh*SOX7*-2+3) or control construct (Scramble). Data are shown as the mean of absolute cell number from three wells (n=3, * *P*<0.05). Graphs are representative of two independent experiments.

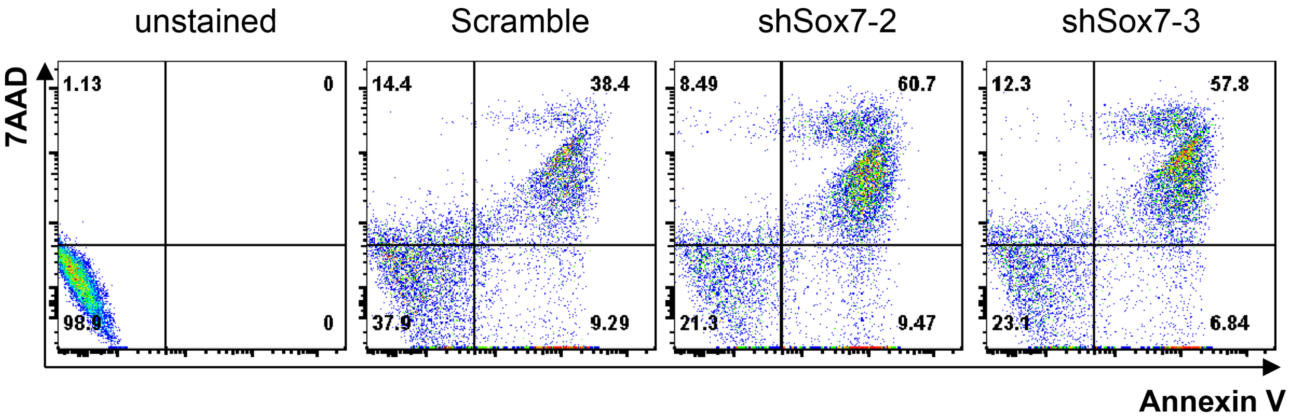

Supplementary Figure S3: Positive control of Annexin V and 7AAD staining. NALM6 human leukemia cells transduced with scramble or shRNAs against *SOX7* were treated with staurosporine (Sigma) at 2mM for 6 hours then stained for Annexin V and 7AAD and analysed by flow cytometry. Data are representative of two independent experiments.

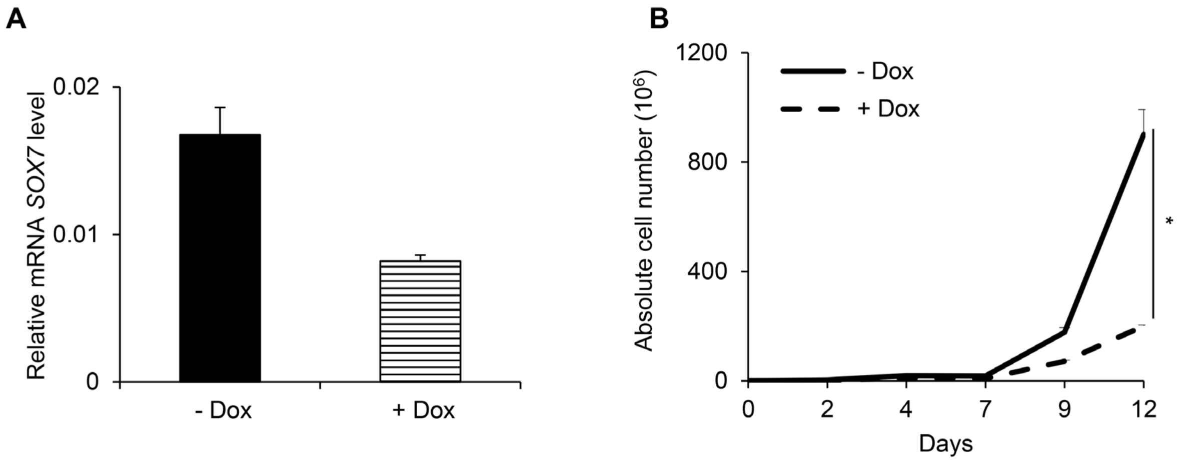

Supplementary Figure S4: Transduction with inducible shRNA does not improve the knock-down of *SOX7*. A. Quantitative RT-PCR analysis of *SOX7* transcript level relative to β2microglobulin in NALM6 human leukemia cells transduced with the inducible shRNAs against *SOX7*. Leukemia cells not treated with doxycycline were used as a positive control. Error bars indicate mean ± standard deviation (n=3). **B.** Proliferation assay performed with NALM6 human leukemia cells transduced the inducible shRNAs against *SOX7*. Data are shown as the mean of absolute cell number from three wells (* $P<0.05$). Data are representative of three independent experiments.
